# Mitochondrial pyruvate carrier function is negatively linked to Warburg phenotype *in vitro* and malignant features in esophageal squamous cell carcinomas

**DOI:** 10.18632/oncotarget.13717

**Published:** 2016-11-30

**Authors:** Yaqing Li, Xiaoran Li, Quancheng Kan, Mingzhi Zhang, Xiaoli Li, Ruiping Xu, Junsheng Wang, Dandan Yu, Mariusz Adam Goscinski, Jian-Guo Wen, Jahn M. Nesland, Zhenhe Suo

**Affiliations:** ^1^ Department of Oncology, the First Affiliated Hospital of Zhengzhou University, Zhengzhou, 450052, Henan Province, China; ^2^ Department of Pathology, the Norwegian Radium Hospital, Oslo University Hospital, University of Oslo, Oslo, 0379, Norway; ^3^ Department of Pathology, the Institute of Clinical Medicine, Faculty of Medicine, University of Oslo, Oslo, 0379, Norway; ^4^ Department of Clinical Pharmacology, the First Affiliated Hospital of Zhengzhou University, Zhengzhou, 450052, Henan Province, China; ^5^ Department of Oncology, the Anyang Tumor Hospital, Anyang, 455000, Henan Province, China; ^6^ Department of Surgery, the Norwegian Radium Hospital, Oslo University Hospital, University of Oslo, Oslo, 0379, Norway; ^7^ Institute of Clinical Medicine, the First Affiliated Hospital of Zhengzhou University, Zhengzhou, 450052, Henan Province, China

**Keywords:** UK5099, Warburg effect, metabolic reprogramming, bioenergetic profiles, HIF-1α

## Abstract

Aerobic glycolysis is one of the emerging hallmarks of cancer cells. In this study, we investigated the relationship between blocking mitochondrial pyruvate carrier (MPC) with MPC blocker UK5099 and the metabolic alteration as well as aggressive features of esophageal squamous carcinoma. It was found that blocking pyruvate transportation into mitochondria attenuated mitochondrial oxidative phosphorylation (OXPHOS) and triggered aerobic glycolysis, a feature of Warburg effect. In addition, the HIF-1α expression and ROS production were also activated upon UK5099 application. It was further revealed that the UK5099-treated cells became significantly more resistant to chemotherapy and radiotherapy, and the UK5099-treated tumor cells also exhibited stronger invasive capacity compared to the parental cells. In contrast to esophageal squamous epithelium cells, decreased MPC protein expression was observed in a series of 157 human squamous cell carcinomas, and low/negative MPC1 expression predicted an unfavorable clinical outcome. All these results together revealed the potential connection of altered MPC expression/activity with the Warburg metabolic reprogramming and tumor aggressiveness in cell lines and clinical samples. Collectively, our findings highlighted a therapeutic strategy targeting Warburg reprogramming of human esophageal squamous cell carcinomas.

## INTRODUCTION

Pyruvate is a central metabolite, which links cytoplasmic and mitochondrial energy metabolism. The fate of pyruvate is one of the most important metabolic decisions made by eukaryotic cells [1]. Cancer cells, however, prefer to divert pyruvate and its precursors to fuel other anabolic processes or convert it to lactate for excretion instead of mitochondrial oxidative phosphorylation (OXPHOS) [2]. This metabolic adaptation was first described by the eminent biochemist Otto Warburg in the 1920s and was termed as “aerobic glycolysis” or Warburg effect [3]. Multiple mechanisms contribute to this metabolic derangement in cancer cells, of which the metabolism of pyruvate plays a central role [4]. Aerobic glycolysis provides bioenergetic intermediates, but generates less ATP. The high concentration of lactate acid produced by the aerobic glycolysis in tumor cells acidifies the extracellular microenvironment, promoting invasion and metastasis, reducing drug efficacy and evading immune recognition [5–7]. It is indicated that the increased glycolytic flux is a metabolic strategy of tumor cells to ensure survival and growth in nutrient-deprived environments [8].

The mitochondrial pyruvate carrier (MPC), which locates in the inner mitochondrial membrane, is strategically positioned at the intersection between cytosolic glycolysis and mitochondrial OXPHOS metabolic processes [9–12]. The MPC complex comprises two paralogous subunits in animal/human, namely MPC1 and MPC2. MPC1 is approximately 12kDa with two predicted transmembrane domains while MPC2 is slightly larger at 14kDa and contains three predicted transmembrane domains. Loss of either MPC1 or MPC2 protein results in the destabilization and degradation of the other and thus loss of the MPC complex function [13].

Recent investigations have demonstrated that the MPC may be a key point of altered metabolic regulation in cancer. The loss of MPC activity in models of cancer promoted a Warburg-like metabolism that could contribute to oncogenic transformation [14–16]. It was also showed that the expression of MPC1 was inhibited or downregulated in multiple cancers and correlated with poor prognosis, including colon, kidney, lung, bladder and brain [14]. Re-expression of MPC1 *in vitro* was able to repress the Warburg effect in colon cancer cells. These authors proposed that reduced MPC activity was an important aspect of cancer metabolism, perhaps through altering the maintenance and fate of cancer stem cells [14]. In addition, it has been verified that UK5099 and α-cyano-4-hydroxycinnamic acid are specific chemical inhibitors of MPC [17].

Given the central metabolic node occupied by the MPC, the change in MPC activity may profoundly regulate overall cellular metabolism. In our present study, we used the specific MPC inhibitor UK5099 to treat a panel of esophageal squamous carcinomas cell lines EC109, KYSE140 and KYSE450 and found that pharmaceutical inhibition of MPC activity dramatically suppressed OXPHOS, induced the Warburg effect and the aggressive cancer phenotype in esophageal squamous cancer cells. We also showed that hypoxia-inducible factor 1α (HIF1α) is occupied in the metabolic and biological switch. In addition, we also determined the expression status of MPC1 and MPC2 in a series of 157 esophageal squamous cell carcinomas and found that the low expression of MPC1 predicted an unfavorable outcome, indicating the regulation of metabolic reprogramming by MPC1 is pivotal for tumor cell growth.

## RESULTS

### MPC1 and MPC2 protein expression in squamous esophageal cancer cells

**Figure 1:**
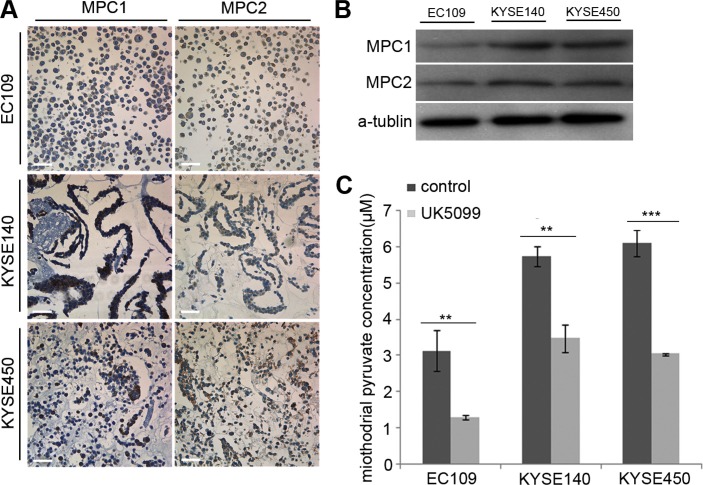
Determination of MPC1 and MPC2 expression and UK5099 blocking effect on pyruvate mitochindrial transportation in squamous esophageal cancer cells (**A**) MPC1 and MPC2 protein expression in esophageal squamous EC109, KYSE140 and KYSE450 cells by ICC assay. (**B**) MPC1 and MPC2 protein expression by Western blot. (**C**) UK5099 treatment reduces the mitochondrial pyruvate concentration in esophageal squamous EC109, KYSE140 and KYSE450 cells *in vitro*. Data are expressed as mean ± SD, *n* = 3.*p < 0.05, **p < 0.01, ***p < 0.001. The scale bar is 50 μm.

To select cancer cell lines expressing MPC1 and MPC2, immunocytochemiscal assay was used to screen the expression status in EC109, KYSE140 and KYSE450 cancer cells. Variable levels of MPC1 and MPC2 protein expressions were identified in these cells, although EC109 cells expressed lower levels of these proteins (Figure 1A), which was also verified by the Western blotting technology (Figure 1B).

### 40 μM UK5099 efficiently blocked pyruvate mitochondrial transportation *in vitro*

In this study, we firstly optimized the UK5099 concentration based on cell viability, mitochondrial pyruvate concentration and lactic secretion. It was discovered that application of 40 μM UK5099 significantly reduced the pyruvate concentration in mitochondria and induced lactic secretion in media but had no obvious effect on the cell viability in the EC109, KYSE140 and KYSE450 cells, the pyruvate concentration in mitochondria of the three cell lines was shown in Figure 1C. It was therefore determined to apply 40 μM UK5099 for further studies in this project.

### UK5099 application stimulated glycolytic metabolism transition

**Figure 2:**
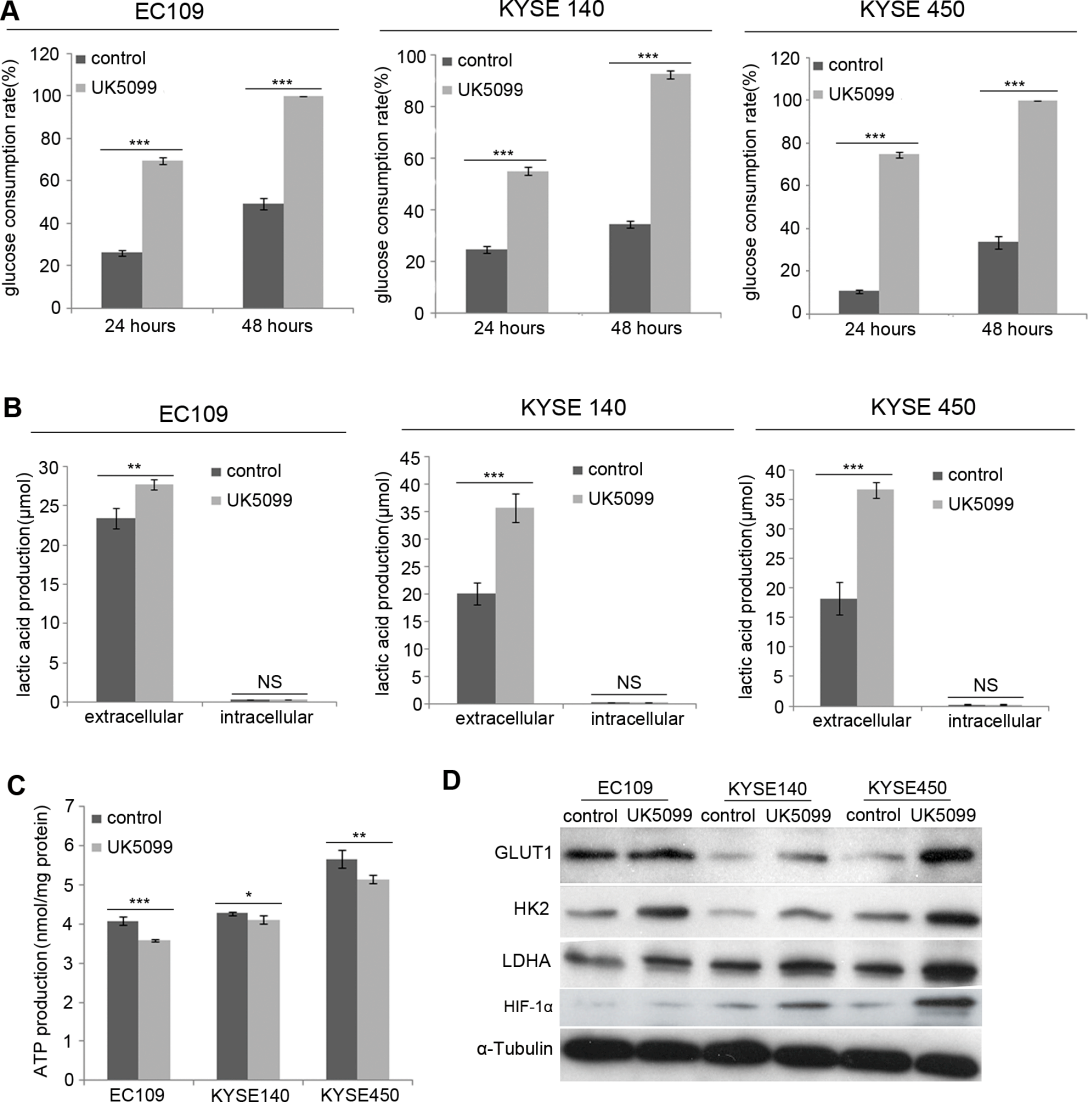
UK5099 treated cells show increased aerobic glycolysis (**A**) EC109, KYSE140 and KYSE450 cells with UK5099 treatment exhibit an increased glucose consumption, both at the time point of 24 hours (*p* < 0.001 for all) and 48 hours (*p* < 0.001 for all) culture. (**B**) UK5099 application induces the lactate acid excretion. Lactate levels in the extracellular media increase in the groups of UK5099 treatment as compared with control groups, while no significant change of lactate is observed in the intracellular lysates. (**C**) UK5099 treated cells produce significant less ATP compared to the control cells. (**D**) The key enzymes of glycolysis pathway GLUT1, HK2, and LDHA in the cells treated with UK5099 are increased. The α-Tubulin serves as a loading control. The results are expressed as mean ± SD, *n* = 3.*p < 0.05, **p < 0.01, ***p < 0.001.

Compared to the control cells, the EC109, KYSE140 and KYSE450 cancer cells treated with UK5099 exhibited significantly increased glucose consumption, both at the time points of 24 hours and 48 hours (Figure 2A, *p* < 0.001 for all three cell lines) culture. Then L-lactatic acid assay revealed that extracellular lactic acid concentration increased significantly in the UK5099 treated EC109, KYSE140 and KYSE450 cells (Figure 2B, *p* = 0.007, 0.001 and 0.000, respectively), compared to the control cells. However, the quantity of the intracellular lactic acid was almost not influenced in all these three cell lines (Figure 2B, *p* = 0.121, 0.081 and 0.878, respectively), indicating significantly higher levels of lactic acid efflux in the cells treated with UK5099. At the meantime, the ATP production in the UK5099 treated EC109, KYSE140 and KYSE450 cells was discovered significantly lower (Figure 2C, *p* = 0.000, 0.013 and0.002, respectively). Afterward, to explore whether UK5099 treatment could drive glycolysis through upregulating the key glycolytic enzymes expression of the glycolysis pathway, glucose transporter 1 (GLUT1), hexokinase II (HK2) and lactate dehydrogenase A (LDHA) expressions were determined by Western blotting. As shown in Figure 2D, UK5099 application resulted in apparently higher levels GLUT1, HK2 and LDHA protein expressions in the treated tumor cells compared to the control cells. In addition, the HIF-1α was also upregulated upon attenuated pyruvate transportation into mitochondria.

### UK5099 application resulted in bioenergetics transition

**Figure 3:**
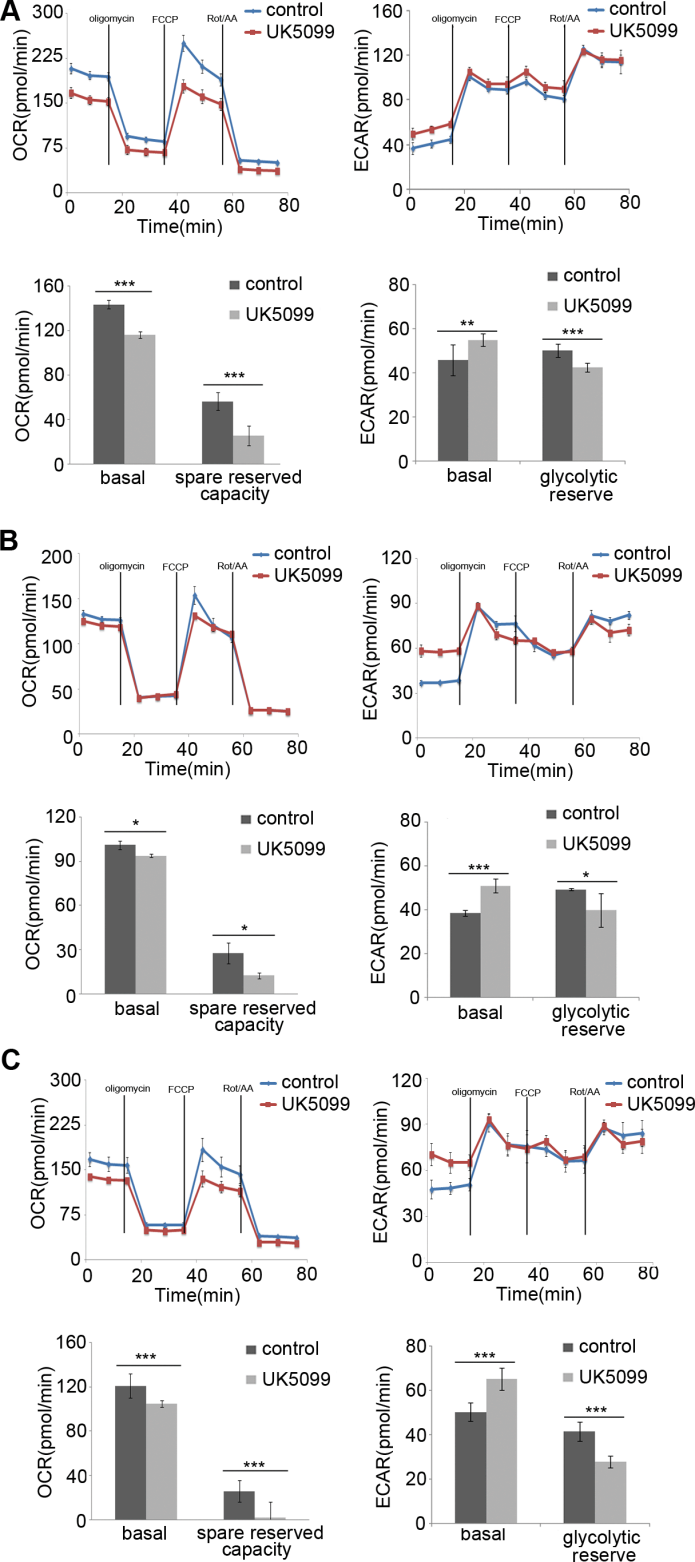
OCR and ECAR The EC109 (**A**), KYSE140 (**B**) and KYSE450 (**C**) cells treated with UK5099 exhibit lower basal respiration and reduced spared reserved respiration capacity while higher basal ECAR and lower glycolytic reserved capacity compared to the control cells. Rot/AA: rotenone and antimycin A. Data are expressed as mean ± SD, *n* = 5.*p < 0.05,**p < 0.01,***p < 0.001.

The mitochondrial bioenergetic profiles in the basal state and after addition of oligomycin, FCCP and rotenone+antimycinA were determined in real time. Oligomycin application was to block ATP synthesis by inhibiting ATP synthase, FCCP was to uncouple ATP synthesis from the flow of electrons in the electron transport chain (ETC) and rotenone+antimycinA was to block ETC complexes I and III, respectively. The concentrations of oligomycin, FCCP, rotenone and antimycin A were firstly optimized for each cell line. Following 1μM oligomycin application, the UK5099 treated cells showed lower ATP-linked O2 consumption and higher proton leakage (Figure 3). Although the maximum ECAR of the two groups was almost the same, the glycolytic reserved capacity of the UK5099 treated cells was significantly lower (Figure 3). In the presence of FCCP, a significant increase of OCR was observed in the control cells, while there was only a slight or moderate increase in the UK5099 treated cells. The increase was considered as respiratory reserve capability. This implied that the UK5099 treated cells were operating lower basic OCR capacity and lower reserved OCR capacity (Figure 3), suggesting defective mitochondrial OXPHOS function and increased glycolytic efflux in the UK5099-treated esophageal squamous cancer cells.

### UK5099 application contributed to perturbed redox homeostasis

**Figure 4:**
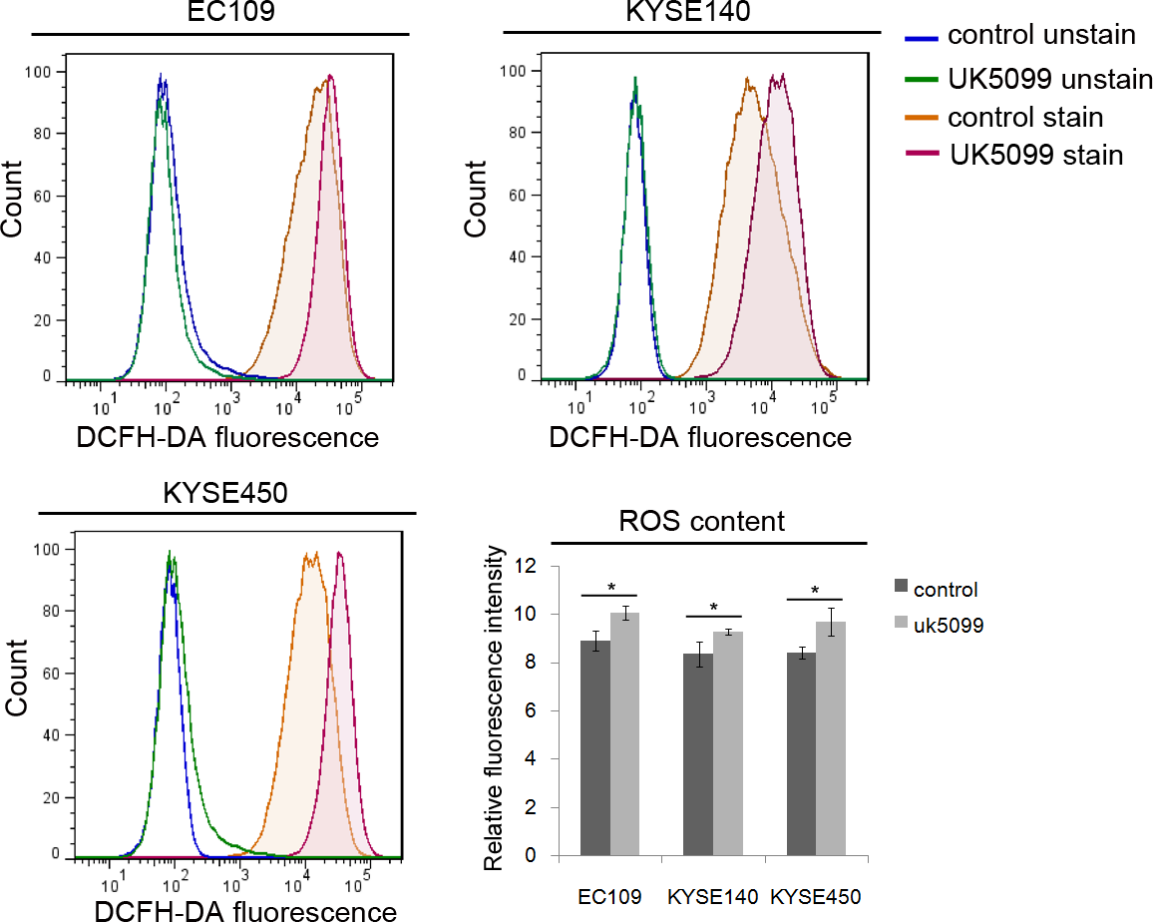
Relatively higher level of ROS in UK5099 treated cells Flow cytometric histograms of ROS levels were determined by DCFDA fluorescence. Relative fluorescence intensity for DCFDA fluorescence was determined from triplicate experiments with SD and *p* values (*t* test) shown. *p < 0.05.

To investigate whether mitochondria function was influenced when imported pyruvate into mitochondria was inhibited by UK5099, the ROS production in esophageal squamous cancer cells was detected. As shown in Figure 4, applications of 40 μM UK5099 in EC109, KYSE140 and KYSE450 cells significantly increased the production of ROS (*p* = 0.017,0.039 and 0.026,respectively), indicating potential possibility that ROS production from the perturbed mitochondrial electron transport chain or decreased antioxidant activity.

### UK5099 application induced higher therapeutic resistance

**Figure 5:**
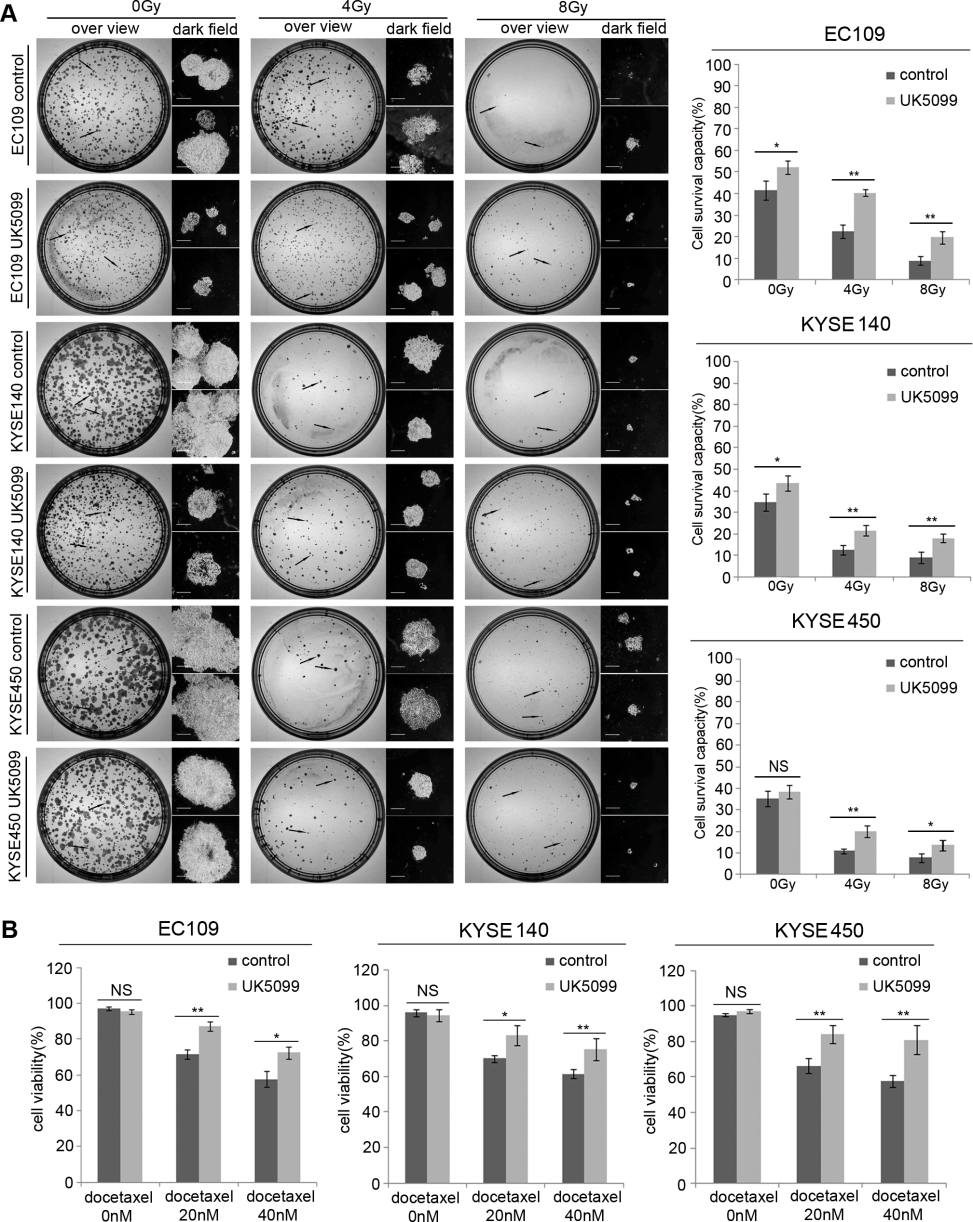
The application of UK5099 application induces therapy resistance in the esophageal squamous cancer cells UK5099 treated esophageal squamous cancer cells exhibit significantly more resistant to the radiotherapy (**A**) and docetaxel treatments (**B**) compared to the control cells. The results are expressed as mean ± SD, *n* = 3.*p < 0.05,**p < 0.01,***p < 0.001. The scale bar is 400 μm.

As shown in Figure 5A, without x-ray irradiation, more UK5099 treated EC109 and KYSE140 cells (*p* = 0.027 and *p* = 0.044, respectively)survived the assay, compared to the control cells. Although the KYSE450 cells did not show higher survival capability (*p* = 0.298) compared to the control cells as the above two cell lines, a similar tendency was also observed as shown in the Figure 5A. There were 40.33% and 19.67% of the EC109 cells treated with UK5099 survived the 4 and 8Gy irradiations while only 22.67% (*p* = 0.001) and 9.00% (*p* = 0.007) control cells survived the irradiations, respectively (Figure 5A). There were 21.67% and 18.00% KYSE140 cells treated with UK5099 survived the 4 and 8Gy irradiations, but only 12.67% % (*p* = 0.009) and 9.00% (*p* = 0.009) of the control cells survived the same irradiations, respectively. Similarly, there were 20.0% (*p* = 0.005) and 13.67% (*p* = 0.038) of the KYSE450 cells treated with UK5099 survived the 4 and 8Gy irradiations while just 11.0% (*p* = 0.005) and 8.0% (*p* = 0.038) of the control cells survived the same irradiations, respectively (Figure 5A).

As shown in Figure 5B, all the cells showed decreased cell viability when docetaxel concentration was increased from 20 nM to 40 nM. However, UK5099 treated cells always displayed significantly increased cell viability compared to the control cells upon docetaxel application. Upon application of 20nM docetaxel, the cell viability was 87.00%, 83.00% and 84.33% for the EC109, KYSE140 and KYSE 450 cells treated with UK5099, while the 71.67% (*p* = 0.002), 70% (*p* = 0.019) and 66.33% (*p* = 0.008) in the control cells, respectively. For the cells treated with 40nM docetaxel, the cell viability was 72.33%, 75.33% and 81.00% for the EC109, KYSE140 and KYSE 450 cells treated with UK5099, while the 57.67% (*p* = 0.011), 61.33% (*p* = 0.021) and 57.67% (*p* = 0.01) in the control cells, respectively.

### UK 5099 treated esophageal squamous cancer cells were highly migratory

**Figure 6:**
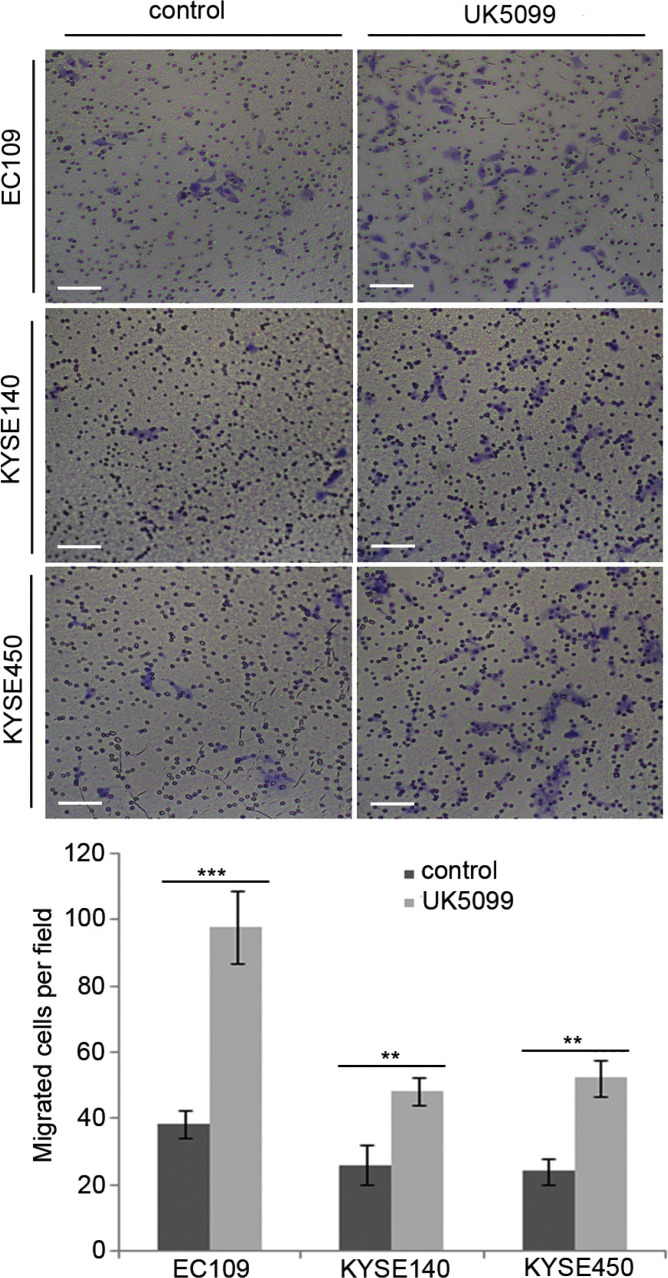
UK5099 application promotes cell migration in the esophageal squamous cancer cells The numbers of the migratory cells are significantly higher in the UK5099 treatment groups than that in the control groups. The results are expressed as mean ± SD, *n* = 3 *p < 0.05, **p < 0.01,***p < 0.001.

It was verified in this study that inhibition of MPC by UK5099 in EC109 cells led to a 157% migration increase compared to the control cells (Figure 6, *p* = 0.001). For the KYSE140 cell line, the UK5099 treatment led to an 86% migration increase compared to the control cells (Figure 5, *p* = 0.006). For theKYSE450 cell line, the UK5099 treatment led to a 118% migration compared to the control cells (Figure 6, *p* = 0.002). All these results indicated a strong migration induction upon the MPC blocking by UK5099 in esophageal squamous cancer cells *in vitro*.

### The MPC1 and MPC2 protein expression in ESCCs

**Figure 7:**
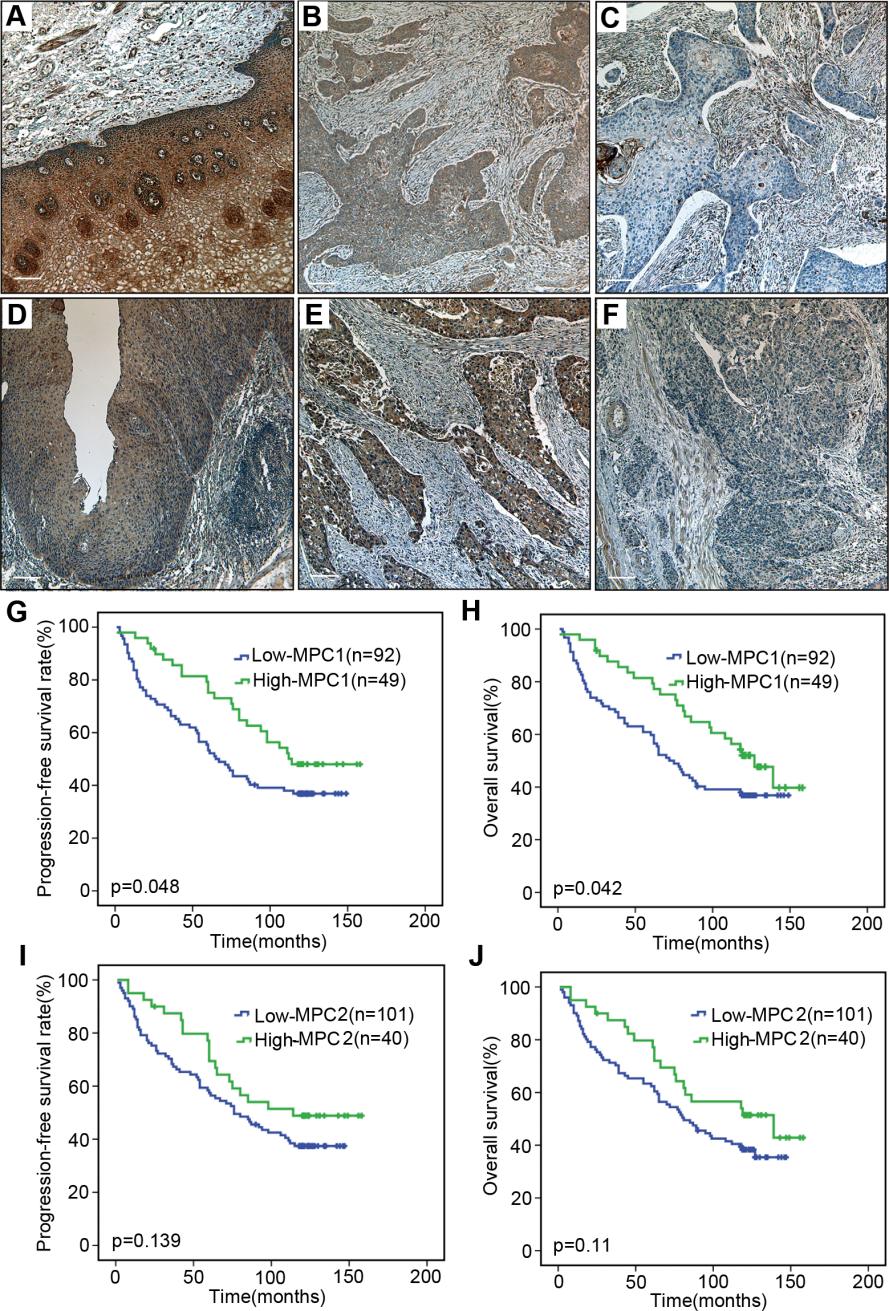
Immunohistochemical assay and survival analyses Positive expression of MPC1 in normal esophageal epithelium (**A**). Representative high (**B**) and low (**C**) expression of MPC1 protein in ESCC tissue. Positive expression of MPC2 in normal esophageal epithelium (**D**). Representative high (**E**) and low (**F**) expression of MPC2 protein in ESCC tissue. The stromal fibroblasts, endothelial cells, smooth muscle cells and infiltrating lymphocytes inside the tumors are always positive for MPC1 and MPC2 expression. The scale bar is 100 μm. Progression-free survival (**G**) and overall survival (**H**) curves according to MPC1 expression in 141 ESCCs. Progression-free survival (**I**) and overall survival (**J**) curves according to MPC2 expression in 141 ESCCs.

The MPC1 and MPC2 protein expressions were detected in a series of 157 ESCC samples. As shown in Figure 7, cytoplasmic MPC1 and MPC2 proteins were highly expressed in 10 normal esophageal epithelia adjacent to tumor. In contrast, MPC1 and MPC2 expressions were aberrantly detected in the tumors. We divided the 157 cases into the low MPC1 or MPC2 expression (score 1–4) and high MPC1 or MPC2 expression (score 6–9). It was discovered that a large number of tumors had negative or low expression of these proteins. In total, 103 of the tumors showed low MPC1 expression (65.6%), while 111 of the tumors were low MPC2 expression (70.7%) (Figure 7A–7F). In addition to this, MPC1 and MPC2 protein expression were also found in the stromal fibroblasts, endothelial cells, smooth muscle cells and infiltrating lymphocytes inside the tumors.

### Decreased expression of MPC protein correlated to advanced tumor stage and distant metastasis

The association between MPC1 and MPC2 protein expressions and the clinicopathological features were analyzed. As summarized in Table 1, MPC1 expression was significantly associated with the depth of tumor invasion, clinical stage and distant metastasis of ESCC (Table 1). No significant association was found between the MPC1 protein expression and other clinical parameters such as age, tumor size, differentiated grade and lymph node metastasis. As for MPC2 protein espression results, which are shown in Table 2, low expression of MPC2 was also associated with the depth of tumor invasion, although no significant difference was observed with other clinical characters.

**Table 1:** Association between MPC1 protein expression and clinicopathological parameters in 157 ESCCs

	Total	MPC1 expression		*p* value
		Low	High	
Ages				0.281
≥ 57	82	57 (69.5%)	25 (30.5%)	
< 57	75	46 (61.3%)	29 (38.7%)	
Sex				0.358
Male	95	65 (68.4%)	30 (31.6%)	
Female	62	38 (61.3%)	24 (38.7%)	
Histology				0.777
Well	56	36 (64.3%)	20 (35.7%)	
Moderate	47	31 (66.0%)	16 (34.0%)	
Poor	51	36 (70.6%)	15 (29.4%)	
Tumor size (mm)				0.674
< 30	30	19 (63.3%)	11 (36.7%)	
30–60	105	69 (65.7%)	36 (34.3%)	
> 60	13	10 (76.9%)	3 (23.1%)	
Tumor depth				0.000^***^
T1/T2	58	24 (41.4%)	34 (58.6%)	
T3/T4	99	79 (79.8%)	20 (20.2%)	
Clinical stage				0.000^***^
I–II	101	56 (55.4%)	45 (44.6%)	
III–IV	46	39 (84.8%)	7 (15.2%)	
Lymph node metastasis				
Yes	95	69 (72.6%)	26 (27.4%)	0.022^*^
No	62	34 (54.8%)	28 (45.2%)	
Distant metastasis				
Yes	38	30 (78.9%)	8 (21.1%)	0.038^*^
No	111	67 (60.4%)	44 (39.6%)	

* *p* < 0.05;

*** *p* < 0.001.

**Table 2:** Association between MPC2 protein expression and clinicopathological parameters in 157 ESCCs

	Total	MPC2 expression		*p* value
		Low	High	
Ages				0.477
≥ 57	82	60 (73.2%)	22 (26.8%)	
< 57	75	51 (68.0%)	24 (32.0%)	
Sex				0.437
Male	95	65 (68.4%)	30 (31.6%)	
Female	62	46 (74.2%)	16 (25.8%)	
Histology				0.677
Well	56	38 (67.9%)	18 (32.1%)	
Moderate	47	35 (74.5%)	12 (25.5%)	
Poor	51	38 (74.5%)	13 (25.5%)	
Tumor size (mm)				0.897
< 30	30	21 (70.0%)	9 (30.0%)	
30–60	105	76 (72.4%)	29 (27.6%)	
> 60	13	10 (76.9%)	3 (23.1%)	
Tumor depth				0.004^**^
T1/T2	58	33 (41.4%)	25 (58.6%)	
T3/T4	99	78 (78.8%)	21 (21.2%)	
Clinical stage				0.129
I–II	101	69 (68.3%)	32 (31.7%)	
III–IV	46	39 (80.4%)	7 (19.6%)	
Lymph node metastasis				
Yes	95	71 (74.7%)	24 (25.3%)	0.169
No	62	40 (64.5%)	22 (35.5%)	
Distant metastasis				
Yes	38	30 (78.9%)	8 (21.1%)	0.257
No	111	77 (69.4%)	34 (30.6%)	

** *p* < 0.01.

### Decreased MPC1 protein expression was associated with poor progression free survival and overall survival

To evaluate the association of MPC1 and MPC2 protein expressions and survival in 141 ESCC patients with survival data, the progression free and overall survival were calculated by the Kaplan-Meier method and compared using the log-rank test. Progression-free survival was defined as the time from surgery until objective assessment of disease progression according to the RECIST guidelines or death. The analysis showed that median overall survival times in the MPC1 high group and in the MPC1 low group were 127.0 months (95% CI 102.1–151.9) and 72.0 months (95% CI 56.02–87.9), respectively (*p* = 0.042) (Figure 7G and 7H). The median progression-free survival times in the MPC1 high group and in the MPC1 low group were 114.0 months and 65.0 months (95% CI 50.5–79.6), respectively (*p* = 0.042). MPC1 protein expression in these ESCC tumors was significantly associated with better progression-free survival and overall survival. Although the median progression-free survival and overall survival of the MPC2 low group were lower (Figure 7I and 7J), there was no significant difference for MPC2 protein expression in both progression-free survival and overall survival.

In addition, multivariate analysis was performed to determine independent prognostic factors for overall survival in esophageal ESCC patients. Our results indicated that even though MPC1 expression had a correlation between the overall survival in the univariate (*p* = 0.042), but the multivariate analysis showed that MPC1 expression was not an independent factor of overall survival for the ESCC patients (Table 3).

**Table 3:** Univariate and multivariate analysis of MPC1 expression for OS in a total of 157 ESCCs

Characteriscs	Unvariate analysis			Multivariate analysis		
	HR	CI (95%)	*p* value	HR	CI (95%)	*p* value
MPC1 expression	0.618	0.386–0.990	0.045^*^	0.934	0.553–1.577	0.797
Tumor depth	2.096	1.301–3.377	0.002^**^	1.619	0.915–2.866	0.098
Histology grade	1.337	1.033–1.729	0.027^*^	1.195	0.915–1.562	0.191
Clinical stage	2.156	1.373–3.386	0.001^**^	1.040	0.440–2.454	0.929
Distant metastasis	2.255	1.418–3.588	0.001^**^	1.659	0.714–3.856	0.239

HR: Hazard ratio; CI: confidence interval.

* *p* < 0.05;

** *p* < 0.01.

## DISCUSSION

Persistent aerobic glycolysis is a key metabolic dependence in carcinogenesis [18]. In our previous study, we have found that MPC complex was a key regulator for glycolysis and mitochondrial OXPHOS and played a critical role in tumor stemness regulation in prostatic cancer cell LNCaP [19]. In this present study, it was verified that upon application of MPC blocker UK5099, the esophageal squamous cancer cells avidly consumed more glucose, generated more lactate acid but less ATP and exhibited significantly lower OCR and higher ECAR. All these results verified that the UK5099 treated cancer cells exhibited decreased mitochondrial OXPHOS and enhanced aerobic glycolysis activity. As decreased oxidative metabolism is often associated with decreased generation of ROS, our results of the relatively higher level of ROS was surprising, but it was consistent with the results of re-expressing MPC in colon cancer cells, which perhaps due to the decreased mitochondrial pyruvate that is known to act as an antioxidant [14, 20].

The contribution of altered glucose metabolism to carcinogenesis and disease progression is currently the focus of intensive research. Gatenby and Gillies proposed that the typical “glycolytic phenotype” of tumor cells conferred a growth advantage and was necessary for the evolution of invasive human cancers, which was also verified by Walenta et al. who found a correlation between lactate concentration in tumor tissues and the incidence of metastases as well as a reduced overall survival of the cancer patients [21–24]. What's more, the glycolytic rate in cultured cell lines also seemed to correlate with tumor aggressiveness. For example, the highly invasive breast cancer cell line MDA-mb-231 exhibited much higher aerobic glucose consumption compared to the non-invasive MCF-7 breast cancer cells [21]. Although the exact mechanism behind this phenomenon was still not fully clear, our study indicated that the HIF1α, which played significant role in the tumor initiation and metastasis, chemoresistance, stemness regulation and metabolism transition [25–29], is one of the potential key regulator between “glycolytic phenotype” and tumor aggressiveness.

Recently, the aberrant expression and activity of enzymes regulating aerobic glycolysis pathway showed crucial roles in tumor progression. There were mounting evidence demonstrating that deregulation of lactate dehydrogenase A (LDHA), which executed the final step of glycolysis by converting the pyruvate to lactate, has been found in many tumors, including prostate cancer, ovarian cancer, breast cancer, osteosarcoma and colorectal cancer, and the upregulated LDHA could facilitate Warburg effect and the progression of tumors [30–35]. Furthermore, increased expression of LDHA was also observed in an osteosarcoma cancer stem cell-like line 3AB-OS, which showed stronger glycolytic metabolism than the parental MG63 cells [36]. In addition, hypoxic tumors, which require increased glycolysis to survive, are often more invasive and metastatic than those less hypoxic [37–39]. Defects in mitochondria, caused by mtDNA depletion or others, can result in metabolic reprogram, which is in accordance with our result. In our group, we found that the prostatic cancer cells lacking mtDNA generated increased levels of lactate with concomitantly reduced oxygen consumption and ATP production, and those mtDNA depletion cells exhibited an increased stemness and malignant phenotype including chemoresistance, radiotherapy resistance and increased migration [40]. All these results have demonstrated the clinical importance of glucose metabolism and have moved the glycolytic phenotype from a laboratory oddity to the mainstream of clinical oncology.

There is a universal observation of aerobic glycolysis in invasive human cancers. Its persistence can be discovered even under normoxic conditions and correlated with tumour aggressiveness, indicating that the glycolytic phenotype may confer a significant proliferative advantage during somatic evolution of cancer. Therefore, aerobic glycolysis of tumor cells is probably a crucial component of the malignant phenotype. The malignant phenotype of ESCCs can be attributed to altered Warburg effect, which provides cancer cells with acidified microenvironment, a factor playing critical role in the initiation and progression of tumor [41]. During the metabolism transition, some factors such as HIF1α and ROS can also induce the malignant phenotype of cancers. Consistent with that, we also discovered that efficient blocking of MPC created the glycolytic phenotype, conferring the cells a dramatically increased survival and invasion advantage as revealed by the experiments of therapy-resistance and transwell capability. Given the importance of Warburg effect in tumor growth and metastasis, we hypothesized that the role of MPC in tumor aggressiveness in ESCC might dependent on the altered Warburg reprogramming, a mechanism involved in remodeling of extracellular matrix due to acidification microenvironment and altered energy metabolism, hereafter a series of imbalance of oncogenic and tumor suppressor factors. The expression of MPC1 was negatively correlated with the MYC oncogene and positively correlated with the colon tumor suppressor APC also supported this hypothesis [14].

In conclusion, we have shown that the inhibition of MPC by UK5099 enforces the esophageal squamous cancer cells to switch toward aerobic glycolysis, typical Warburg reprogramming, and endorses the cells with significantly higher survival capability against chemotherapy and radiotherapy, in addition to invasion and metastasis advantages. The fact that suppression or under-expression of MPC in ESCC tissue specimens was associated with cancer onset, clinical metastasis status and poor prognosis is in line with the *in vitro* findings. Therefore, placing the MPC properly in the metabolic network of cancer cells might supply a more sophisticated and specific approach for therapeutic benefit.

## MATERIALS AND METHODS

### Cell lines and culture conditions

Esophageal squamous caner KYSE140 and KYSE450 cell lines were obtained from ATCC (American Type Culture Collection, USA) and maintained in our laboratory for the study. The EC109 cell line was purchased from Chinese Academy of Medical Sciences (Shanghai, China). Cells were cultured in RPMI 1640 (Gibco™, 11835-063) supplemented with 10% fetal bovine serum (Gibco™, 10500–064), 100 U/ml of penicillin and 100 ug/ml streptomycin (Life Technologies, 15140122) at 37°C and 5% CO2. UK5099(PZ0160, Sigma-Aldrich, St. Louis, MO,USA), was optimized to a final concentration of 40 μM to reduce pyruvate transportation into mitochondrial based on a series of UK5099 dose tested in a range of 10 μM to 100 μM as previously published [19].

### Mitochondrial pyruvate measurement

The mitochondria were isolated by performing the Mitochondria/Cytosol Fractionation Kit (BioVision, K256-25, California, USA). Briefly, 1X Cytosol extraction buffer supplemented with DTT and protease inhibitors were added to the cells after 5 × 10^7^ cells were collected and centrifuged. Then the cells were homogenized in an ice-cold dounce tissue grinder (BioVision, 1998-1, California, USA). The mitochondrial fraction was collected after the supernatant was centrifuged after 60 passes. The mitochondrial lysates were collected after the mitochondrial extraction buffer was added to the pellets and vortexed for 10 seconds. Mitochondrial pyruvate concentration was determined by pyruvate assay kit (BioVision, K609-100, California, USA) according to the instructions. Briefly, 50 μl working reagent (including enzyme mix and dye reagent) and 50 μl test sample or standard were mixed and added in 96-well plate. The color intensity of the reaction product at 570 nm was read and recorded with a Biochrom Assays UVM340 Microplate Reader after 30 min's incubation at room temperature. The readout was normalized to the input cell numbers.

### Western blotting

Cells were firstly lysed in RIPA buffer supplemented with a cocktail of protease inhibitors. Cell lysates were centrifuged at 13000 g for 10 minutes at 4°C before aliquots of 20 μg proteins were resolved on SDS-PAGE, transferred onto polyvinylidenedifluoride transfer membrane(PVDF), and blotted for targeting antibodies:MPC1(HPA045119, Sigma-Aldrich, St. Louis, MO,USA, dilution 1:1000), MPC2(ab111380, abcam, Cambridge, UK, dilution 1:1000), GLUT1(Rabbit mAb#12939, cell signaling technology, Leiden, Netherlands, dilution 1:1000), HK2 (ab104836, abcam, Cambridge, UK, dilution 1:1000), LDHA (ab47010, abcam, Cambridge, UK, dilution 1:1000) and α-Tubulin antibody(T9026, Sigma-Aldrich, St. Louis, MO, USA dilution 1:3000).

### Glucose consumption assay

The glucose assay kit (GAHK-20, Glucose Determination kit, Sigma-Aldrich, St. Louis, MO, USA) according to the manufacturer's instruction was used to determine glucose consumption. In brief, 5 × 10^5^ cells/well in 2 ml media cells were seeded in 6-well plates. The media were removed and the concentration of glucose in the medium was measured using a Biochrom Assays UVM340 Microplate Reader after 24 and 48 hours culture. Data were normalized based on the viable cell counts measured by the MTT assays. All the experiments were performed in triplicate and repeated twice.

### Lactate acid assay

The lactate assay was performed using an EnzyChrome^TM^ L-Lactate assay kit (ECLC-100, BioAssay, CA, USA) according to the manufacturer's instructions. Briefly, 5 × 10^5^cells in 2ml medium each well were seeded into 6-well plates and cultured for 24 hours. Then the media and the cell lysates in 200 μl PBS by sonication (in ice-water bath) were centrifuged and collected for the lactate assay. 80 μl working reagents were added to each standard or sample. The lactate concentration was measured at 565 nm using a Biochrom Assays UVM340 Microplate Reader. Experiments were conducted in triplicates and data were normalized based on the different cell division rate.

### Determination of ATP production

1 × 10^6^ cultured cells were harvested in 200 μl PBS and cell lysate was achieved by sonication (in ice-water bath) before the homogenate was centrifuged at 12,000 g for 10 min. The intracellular ATP production was assessed by using a bioluminescence assay (firefly luciferase) ATP Determination Kit (A22066, Molecular Probes™) and the manufacturers' instructions were strictly followed. Luminescence was measured by a luminometer (Fluroskan Ascent FL, Thermo Scientific). Data were normalized based on the protein concentration measured by the Brandford assay. Total ATP levels were expressed as nmol/mg protein.

### OCR and ECAR measurement using seahorse XF^e^96-extracellular flux analyzer

To measure mitochondrial bioenergetics profile, the Seahorse Bioscience XFe96 Extracellular Flux Analyzer was used (Seahorse Bioscience). Briefly, cells were plated at 3 × 10^4^ cells/well in anXFe96 cell culture microplate (Seahorse Bioscience) and cultured overnight to allow the cells to attach to the bottom. The cells were switched into the Seahorse XF Base Medium(Seahorse Bioscience, Part # 102353-100) supplemented with 10 mM glucose,1 mM sodium pyruvate and 2 mM glutamine for at least 1 hour prior to the beginning of the assay and maintained at 37°Cin a CO2-free incubator. OCR and ECAR were reported in the unit of picomoles per minute. After baseline measurements, OCR and ECAR were measured by sequentially adding to each well with 1 μM oligomycin, 0.5 or 0.25 μM carbonylcyanide-p-trifluoromethoxyphenylhydrazone (FCCP) and 0.5 μM rotenone and antimycin A.

### ROS assay

Intracellular ROS production was detected using 2′,7′-dichlorodihydrofluorescein diacetate (DCFH-DA; Molecular Probes) at a final concentration of 5 μM according to the manufacturers protocol. Following treatment, the cells were washed and incubated with DCFH-DA for 20 min at 37°C in the dark. The fluorescence corresponding to the intracellular ROS levels was monitored and analyzed by flow cytometry (BD FACSCalibur flow cytometer, FACS101; BIO-RAD Corporation).

### Radiotherapy

To evaluate radiotherapy response, we performed a modified colony formation assay after subjecting cells to different doses of X-ray irradiation [40]. 3000 cells were seeded into 60mm dishes and were allowed to attach to vessel before X-ray irradiation. Then indicated intensities of X-Ray irradiation were performed onto the dishes in an x-ray irradiator (Faxitron). The colonies and the cells were fixed with methanol and stained with 0.1% (w/v) crystal violet after 2 weeks' cultivation visible. Then the potential colonies were counted in a G: BOX F3 multifunction imaging system with related software (Syngene). The microscopic counting and photograph were carried out on a customized dark-field microscope for the isolated cells which were less than 30 cells. Most importantly, the cell survival capability was calculated in such a way that no matter the colonies contained more and fewer than 30 cells, each colony was explained derived from one single cell.

### Chemotherapy

Cells were seeded in 25 cm flasks at a density of 2 × 10^5^ cells per flask and allowed to attach for 12 hours. Various concentrations (0, 10, 20, 40, 80 nmol/L) of docetaxel were firstly tested, and 20 and 40 nM were selected for further applications in this study. Seventy-two hours after the drug was added to flask for culture, 10 μl of single cell was made from each flask using 0.25% trypsin (Invitrogen) mixed well with 10 μl 0.4% trypan blue dye and counted by Countess^®^ Automated Cell Counter (Life Technologies). Cell viability was calculated based on the survival of non-treated cells. The experiments were conducted in triplicate.

### Cell migration assay

Esophageal squamous cancer cells(2 × 10^4/^/well) were suspended in 200 μl serum-free RPMI 1640 and placed into the upper compartment of the chambers (8 μm pore size, Millipore). Then, 800 μL medium containing 10% fetal bovine serum was added to a 24-well plate. After incubation at 37°C (EC109: 12 hours; KYSE140:16 hours; KYSE450:24 hours), the migratory cells were fixed in methanol and stained with 0.1% crystal violet. Cells were counted using light microscope (Nikon TE300, Tokyo, Japan) at 100× magnification. Five random fields were selected for examination on each membrane, and results were expressed in terms of the migration cells per field and normalized to the averaged numbers under control conditions to assess relative migration capability. Each experiment was conducted in triplicate.

### Patients

The Ethics Committee of Anyang Tumor Hospital and Anyang Hygiene Bureau approved this study. The written informed consents were provided by the patients involved. All experiments were performed in accordance with approved guidelines and regulations. All the patients underwent operation at Anyang Tumor Hospital, Henan, P. R. China between 1989 and 1994 and were followed up from the confirmed date of diagnosis until death or 31 May 2004. Tumors were classified in terms of the International Union against Cancer (UICC) 2003 standard [42]. In addition, 10 esophageal squamous epithelia samples adjacent to tumor were also studied.

### Immunocytochemistry (ICC) and immunohistochemistry (IHC)

Cytoblocks were prepared for ICC and DakoEnVision™ Flex+ (K8012; Dako, Glostrup, Denmark) was applied for ICC and IHC staining as previously described [43]. After deparaffinization, unmasking epitopes and blocking of peroxidase, the slides were then incubated with the following reagents: rabbit monoclonal antibody against human MPC1(HPA045119, Sigma-Aldrich, St. Louis, MO, USA, diluted 1:500) and MPC2(ab111380, abcam, Cambridge, UK, diluted 1:500) at 4°C overnight. Negative controls were produced using the same concentration of non-immune rabbit IgG instead of the rabbit antibody to human MPC1 and MPC2.

### Evaluation of IHC staining

Sections with no labeling or fewer than 5%, 15–25%, 25–50%, and more than 50% labeled cells were scored as 0, 1, 2 and 3, respectively while the staining intensity with negative staining, weakly positive, moderately positive and strongly positive were scored as 0, 1, 2 and 3, respectively. The scores for both the percentage of positive cells and the staining intensity were multiplied to generate an immune-reactive score for each specimen and the scores were finally graded as followings: 0 was graded as no expression, 1–4 was graded as low expression and 6–9 was graded as high expression. Each sample was evaluated by two pathologists. The photos were taken with a Leica DMLB light microscope equipped with SPOT Advanced Software (Olympus, Nagano, Japan).

### Statistical analysis

All data represented at least three independent experiments and statistical analyses were performed using SPSS18.0 version. Data were analyzed by the Student's *t* test(2-tailed) or ANOVA. Chi-square tests (Pearson and linear-by-linear as appropriate) were performed for analyzing the associations between MPC expression and clinicopathological variables. Survival curves were plotted through the Kaplan-Meier method and compared with two-sided log-rank test. A *p value* of less than 0.05 was regarded as statistically significance.

## References

[1] Bowman CE, Zhao L, Hartung T, Wolfgang MJ (2016). Requirement for the Mitochondrial Pyruvate Carrier in Mammalian Development Revealed by a Hypomorphic Allelic Series. Mol Cell Biol.

[2] Vander Heiden MG, Cantley LC, Thompson CB (2009). Understanding the Warburg effect: the metabolic requirements of cell proliferation. Science.

[3] Warburg O, Wind F, Negelein E (1927). The metabolism of tumors in the body. J Gen Physiol.

[4] Bayley JP, Devilee P (2012). The Warburg effect in 2012. Curr Opin Oncol.

[5] Fischer K, Hoffmann P, Voelkl S, Meidenbauer N, Ammer J, Edinger M, Gottfried E, Schwarz S, Rothe G, Hoves S, Renner K, Timischl B, Mackensen A (2007). Inhibitory effect of tumor cell-derived lactic acid on human T cells. Blood.

[6] Gottfried E, Kunz-Schughart LA, Ebner S, Mueller-Klieser W, Hoves S, Andreesen R, Mackensen A, Kreutz M (2006). Tumor-derived lactic acid modulates dendritic cell activation and antigen expression. Blood.

[7] Koukourakis MI, Giatromanolaki A, Sivridis E, Gatter KC, Harris AL (2006). Lactate dehydrogenase 5 expression in operable colorectal cancer: strong association with survival and activated vascular endothelial growth factor pathway—a report of the Tumour Angiogenesis Research Group. J Clin Oncol.

[8] Gatenby RA, Gillies RJ (2008). A microenvironmental model of carcinogenesis. Nat Rev Cancer.

[9] Schell JC, Rutter J (2013). The long and winding road to the mitochondrial pyruvate carrier. Cancer Metab.

[10] Herzig S, Raemy E, Montessuit S, Veuthey JL, Zamboni N, Westermann B, Kunji ER, Martinou JC (2012). Identification and functional expression of the mitochondrial pyruvate carrier. Science.

[11] Halestrap AP (2012). The mitochondrial pyruvate carrier: has it been unearthed at last?. Cell Metab.

[12] Bricker DK, Taylor EB, Schell JC, Orsak T, Boutron A, Chen YC, Cox JE, Cardon CM, Van Vranken JG, Dephoure N, Redin C, Boudina S, Gygi SP (2012). A mitochondrial pyruvate carrier required for pyruvate uptake in yeast, Drosophila, and humans. Science.

[13] Compan V, Pierredon S, Vanderperre B, Krznar P, Marchiq I, Zamboni N, Pouyssegur J, Martinou JC (2015). Monitoring Mitochondrial Pyruvate Carrier Activity in Real Time Using a BRET-Based Biosensor: Investigation of the Warburg Effect. Mol Cell.

[14] Schell JC, Olson KA, Jiang L, Hawkins AJ, Van Vranken JG, Xie J, Egnatchik RA, Earl EG, DeBerardinis RJ, Rutter J (2014). A role for the mitochondrial pyruvate carrier as a repressor of the Warburg effect and colon cancer cell growth. Mol Cell.

[15] Vacanti NM, Divakaruni AS, Green CR, Parker SJ, Henry RR, Ciaraldi TP, Murphy AN, Metallo CM (2014). Regulation of substrate utilization by the mitochondrial pyruvate carrier. Mol Cell.

[16] Yang C, Ko B, Hensley CT, Jiang L, Wasti AT, Kim J, Sudderth J, Calvaruso MA, Lumata L, Mitsche M, Rutter J, Merritt ME, DeBerardinis RJ (2014). Glutamine oxidation maintains the TCA cycle and cell survival during impaired mitochondrial pyruvate transport. Mol Cell.

[17] Hildyard JC, Ammala C, Dukes ID, Thomson SA, Halestrap AP (2005). Identification and characterisation of a new class of highly specific and potent inhibitors of the mitochondrial pyruvate carrier. Biochim Biophys Acta.

[18] Devic S (2016). Warburg Effect - a Consequence or the Cause of Carcinogenesis?. J Cancer.

[19] Zhong Y, Li X, Yu D, Li X, Li Y, Long Y, Yuan Y, Ji Z, Zhang M, Wen JG, Nesland JM, Suo Z (2015). Application of mitochondrial pyruvate carrier blocker UK5099 creates metabolic reprogram and greater stem-like properties in LnCap prostate cancer cells *in vitro*. Oncotarget.

[20] Wang X, Perez E, Liu R, Yan LJ, Mallet RT, Yang SH (2007). Pyruvate protects mitochondria from oxidative stress in human neuroblastoma SK-N-SH cells. Brain Res.

[21] Gatenby RA, Gillies RJ (2004). Why do cancers have high aerobic glycolysis?. Nat Rev Cancer.

[22] Walenta S, Wetterling M, Lehrke M, Schwickert G, Sundfor K, Rofstad EK, Mueller-Klieser W (2000). High lactate levels predict likelihood of metastases, tumor recurrence, and restricted patient survival in human cervical cancers. Cancer Res.

[23] Walenta S, Salameh A, Lyng H, Evensen JF, Mitze M, Rofstad EK, Mueller-Klieser W (1997). Correlation of high lactate levels in head and neck tumors with incidence of metastasis. Am J Pathol.

[24] Walenta S, Schroeder T, Mueller-Klieser W (2004). Lactate in solid malignant tumors: potential basis of a metabolic classification in clinical oncology. Curr Med Chem.

[25] Zhou W, Choi M, Margineantu D, Margaretha L, Hesson J, Cavanaugh C, Blau CA, Horwitz MS, Hockenbery D, Ware C, Ruohola-Baker H (2012). HIF1alpha induced switch from bivalent to exclusively glycolytic metabolism during ESC-to-EpiSC/hESC transition. Embo j.

[26] Prigione A, Rohwer N, Hoffmann S, Mlody B, Drews K, Bukowiecki R, Blumlein K, Wanker EE, Ralser M, Cramer T, Adjaye J (2014). HIF1alpha modulates cell fate reprogramming through early glycolytic shift and upregulation of PDK1–3 and PKM2. Stem Cells.

[27] Hanna SC, Krishnan B, Bailey ST, Moschos SJ, Kuan PF, Shimamura T, Osborne LD, Siegel MB, Duncan LM, O'Brien ET, Superfine R, Miller CR (2013). HIF1alpha and HIF2alpha independently activate SRC to promote melanoma metastases. J Clin Invest.

[28] Zhan M, Wang H, Chen T, Chen W, Yang L, He M, Xu S, Wang J (2015). NOX1 mediates chemoresistance via HIF1alpha/MDR1 pathway in gallbladder cancer. Biochem Biophys Res Commun.

[29] Corcoran SE, O'Neill LA (2016). HIF1alpha and metabolic reprogramming in inflammation. J Clin Invest.

[30] Xian ZY, Liu JM, Chen QK, Chen HZ, Ye CJ, Xue J, Yang HQ, Li JL, Liu XF, Kuang SJ (2015). Inhibition of LDHA suppresses tumor progression in prostate cancer. Tumour Biol.

[31] Qiu H, Jackson AL, Kilgore JE, Zhong Y, Chan LL, Gehrig PA, Zhou C, Bae-Jump VL (2015). JQ1 suppresses tumor growth through downregulating LDHA in ovarian cancer. Oncotarget.

[32] Fantin VR, St-Pierre J, Leder P (2006). Attenuation of LDH-A expression uncovers a link between glycolysis, mitochondrial physiology, and tumor maintenance. Cancer cell.

[33] Zhao YH, Zhou M, Liu H, Ding Y, Khong HT, Yu D, Fodstad O, Tan M (2009). Upregulation of lactate dehydrogenase A by ErbB2 through heat shock factor 1 promotes breast cancer cell glycolysis and growth. Oncogene.

[34] Wang J, Wang H, Liu A, Fang C, Hao J, Wang Z (2015). Lactate dehydrogenase A negatively regulated by miRNAs promotes aerobic glycolysis and is increased in colorectal cancer. Oncotarget.

[35] Gao S, Tu DN, Li H, Jiang JX, Cao X, You JB, Zhou XQ (2016). Pharmacological or genetic inhibition of LDHA reverses tumor progression of pediatric osteosarcoma. Biomed Pharmacother.

[36] Palorini R, Votta G, Balestrieri C, Monestiroli A, Olivieri S, Vento R, Chiaradonna F (2014). Energy metabolism characterization of a novel cancer stem cell-like line 3AB-OS. J Cell Biochem.

[37] Postovit LM, Adams MA, Lash GE, Heaton JP, Graham CH (2002). Oxygen-mediated regulation of tumor cell invasiveness. Involvement of a nitric oxide signaling pathway. J Biol Chem.

[38] He X, Brenchley PE, Jayson GC, Hampson L, Davies J, Hampson IN (2004). Hypoxia increases heparanase-dependent tumor cell invasion, which can be inhibited by antiheparanase antibodies. Cancer Res.

[39] Postovit LM, Adams MA, Lash GE, Heaton JP, Graham CH (2004). Nitric oxide-mediated regulation of hypoxia-induced B16F10 melanoma metastasis. Int J Cancer.

[40] Li X, Zhong Y, Lu J, Axcrona K, Eide L, Syljuasen RG, Peng Q, Wang J, Zhang H, Goscinski MA, Kvalheim G, Nesland JM, Suo Z (2016). MtDNA depleted PC3 cells exhibit Warburg effect and cancer stem cell features. Oncotarget.

[41] Cardone RA, Casavola V, Reshkin SJ (2005). The role of disturbed pH dynamics and the Na+/H+ exchanger in metastasis. Nat Rev Cancer.

[42] Talsma K, van Hagen P, Grotenhuis BA, Steyerberg EW, Tilanus HW, van Lanschot JJ, Wijnhoven BP (2012). Comparison of the 6th and 7th Editions of the UICC-AJCC TNM Classification for Esophageal Cancer. Ann Surg Oncol.

[43] Huang R, Ma Y, Holm R, Trope CG, Nesland JM, Suo Z (2013). Sex hormone-binding globulin (SHBG) expression in ovarian carcinomas and its clinicopathological associations. PLoS One.

